# Local Reasons to Give Globally: Identity Extension and Global Cooperation

**DOI:** 10.1038/s41598-017-15683-0

**Published:** 2017-11-14

**Authors:** Nancy R. Buchan, Sophia Soyoung Jeong, A. K. Ward

**Affiliations:** 1 Department of International Business, Darla Moore School of Business, University of South Carolina, Columbia, SC USA; 2 Management Department, Faculty of Business Administration, Chinese University of Hong Kong, Hong Kong, China; 3 Department of Management, Pamplin College of Business, Virginia Tech University, Blacksburg, VA USA

## Abstract

Recent political events across the world suggest a retrenchment from globalization and a possible increase in parochialism. This inward-looking threat from parochialism occurs just as the global community faces growing challenges that require trans-national cooperation. In this research, we question if strong identification with an in-group necessarily leads to parochialism and ultimately is detrimental to global cooperation. Building on research on global social identification, we explore whether strong local identification can expand in inclusiveness to global identification, and among whom this is likely to happen. The results of our global public goods study – conducted in South Korea and the United States – show that high levels of social identification with a local group can extend to the global collective, particularly for individuals who are also high in concern-for-others. Furthermore, this identification translates into behavior that benefits the global, anonymous group at a cost to oneself. These results shed light on how to avoid the trap of parochialism and instead engender cooperative behavior with the broader global community.

## Introduction

Research supports the notion that humans show favoritism towards members of narrow identity-based in-groups - often to the detriment of out-group members^1–7^. Such parochialism can impede progress where resolution of global dilemmas requires trans-national cooperation, for example, when trying to address problems such as global warming, water shortages, pollution, terrorism and disease^8^. But is strong identification with an in-group always necessarily detrimental to cooperation with a broader group? Here, we explore whether local identification can expand in inclusiveness, and among whom this is likely to happen. The results of our global public goods study – conducted in South Korea and the United States – show that high levels of social identification with a local group can extend to the global collective but that this extension is more likely for individuals who are also high in the trait of concern-for-others. Furthermore, this locally-expanded identification translates into cooperative behavior that benefits the global, anonymous group at a cost to oneself. Among participants who are low in concern-for-others, on the other hand, identification with the local group is unrelated to identification with the global community and to contribution to the global group. These results suggest that understanding the factors that aid in identity extension, such as concern-for-others, may be a key to pushing past parochial boundaries and ultimately, to addressing the challenges we face as a global community.

In recent decades, scholars have discussed how global connectivity may erode parochial ethnic or local identities, thereby promoting a cosmopolitan identity such that “humankind becomes a ‘we’ where there are no ‘others’”^9^. Yet, events such as Brexit, and recent elections internationally suggest a worldwide retrenchment from globalization and a turn toward parochialism, as increasing numbers of people feel their ethnic identity threatened^10^. This retrenchment occurs even as the world suffers acutely from global challenges – from water shortages to combating terrorism to the spread of diseases - whose solutions require interdependent efforts across nations and their citizens^8^. We are thus faced with crucial dilemmas in which cooperation among constituents in the global community is needed just as such cooperation is becoming more difficult to achieve.

Global social dilemmas present individuals with the choice of acting in their own self-interest versus cooperating to promote the collective global welfare. Each individual’s own welfare is maximized by acting selfishly, but if all do so, collective welfare declines^11^. Research suggests that identification with the global community may influence cooperation in such dilemmas. Indeed, in a multi-country public goods game, Buchan and colleagues demonstrated that identification with the global community is a meaningful psychological construct that motivates cooperation beyond parochial self-interest and predicts contributions to a global public good^12^.

The relationship between social identification and cooperation is not new, as a great deal of research has focused on prosocial behaviors towards in-group members. The notion of *parochialism* (favoring members of a narrow, local in-group)^2^ dates back to Darwin’s original writings on human evolution^3^. Research across a variety of fields (e.g., biology, economics, evolution studies, psychology, neuroscience) has consistently shown a tendency for humans to exhibit prosocial and preferential treatment towards those with whom they identify^1,5–7^. Even individualistic, self-focused individuals have been found to make prosocial decisions when in-group identification is strong^13^. A potential dark side to parochialism is that in-group altruism often drives individuals to protect their group through out-group hostility^4,5^.

Given the well-established link between in-group identification and cooperation, the choice between cooperation with a local in-group versus with a collective of global strangers may produce tension that impedes resolution of global challenges, particularly in times of increasing parochialism. Expanding the boundaries of the local community may help to overcome this parochial/global tension. The fundamental questions asked in this study therefore are: is it possible for identification with the local in-group to extend to identification with the global community? Furthermore, does this extension of identification to the global collective translate into global cooperation?

Prior theories and research lead to mixed predictions. When multiple identities are salient simultaneously, two strategies for addressing the identities are available^14^: Additive and conjunctive. An additive strategy increases the inclusiveness of an individual’s identity. According to the Common In-group Identity Model^15^, it is possible to extend one’s identity such that subgroups become nested within a superordinate group. Across a series of studies, Gaertner and colleagues found that individuals can maintain salience of dual identities – for a subordinate and a superordinate group - at once. As a result of such recategorization, individuals exhibit more positive attitudes and behaviors toward former out-group members^16^. In other words, additive strategies suggest that individuals can perceive themselves as belonging to all of humanity without losing their local identities and will likely behave cooperatively toward both groups.

On the other hand, a conjunctive strategy restricts the in-group to the overlap between two groups, in this case, to those who are both local and global - but not nonlocal global - narrowing the boundaries of group identification and potentially increasing the size of the out-group^14^. For example, research on European identity suggests such a resistance to identity extension^17^. According to the Eurobarometer, in 2015 49% of respondents living in European countries were either very or fairly attached to the European Union, while 92% felt such attachment toward their country, and 88% toward their local towns^18^. This suggests that while people may practice an additive strategy between their local and national identification, they seem to use a conjunctive strategy with respect to their identification with the more distal European community. Identification with the proximal community (i.e., locality) was nested within identification to the distal community (i.e., European Union) for only half the respondent population even after almost sixty years of efforts to integrate the countries through a common market system.

We are thus compelled to ask, when do individuals with strong local identification extend this sense of connection and belonging to a broader community, and can this identity extension translate into cooperation with the broader community as well? We explored participants’ *concern-for-others -* an individual’s value for being helpful towards other people^19^ – as a trait characteristic that may make individuals more susceptible to identity extension and universal cooperation. Drawing upon Simon’s theory of altruism^20,21^, researchers have shown that individuals valuing concern-for-others tend to be focused on developing a sense of belonging or connectedness^22–24^. Thus, we ask whether concern-for-others aides in the identity extension process, such that those high in this trait are more likely than others to seek such connectedness at all levels, and if this extension leads to enhanced global cooperation.

We studied these questions in a social dilemma consisting of local and global public goods games conducted in South Korea and the United States in which we also measured participants’ local and global social identification. Participants were given the opportunity to keep tokens with monetary value for themselves or to contribute them to a group of anonymous players – either from their in-group or from the global community - in which case they could benefit the group of others at an expense to themselves.

## Results

### Identity Extension

We first explored whether local social identification would extend to identification with the global community and whether this effect was moderated by concern-for-others. We conducted a multiple regression analysis testing the interaction effect between local social identification and concern-for-others on global social identification. The model including country, income, local social identification, and concern-for-others (adjusted R^2^ = 0.16, *F*(4, 168) = 8.21, *p* < 0.001) showed a positive direct effect of local social identification on global social identification (*b* = 0.33, *SE* = 0.07, 95% CI = [0.19, 0.47], *t*(168) = 4.77, *p* < 0.001), indicating that social identification with the local community was positively related to global social identification. Concern-for-others did not predict global social identification (*b* = 0.11, *SE* = 0.07, 95% CI = [−0.03, 0.24], *t*(168) = 1.51, *p* = 0.134).

### Concern-for-Others

**Figure 1:**
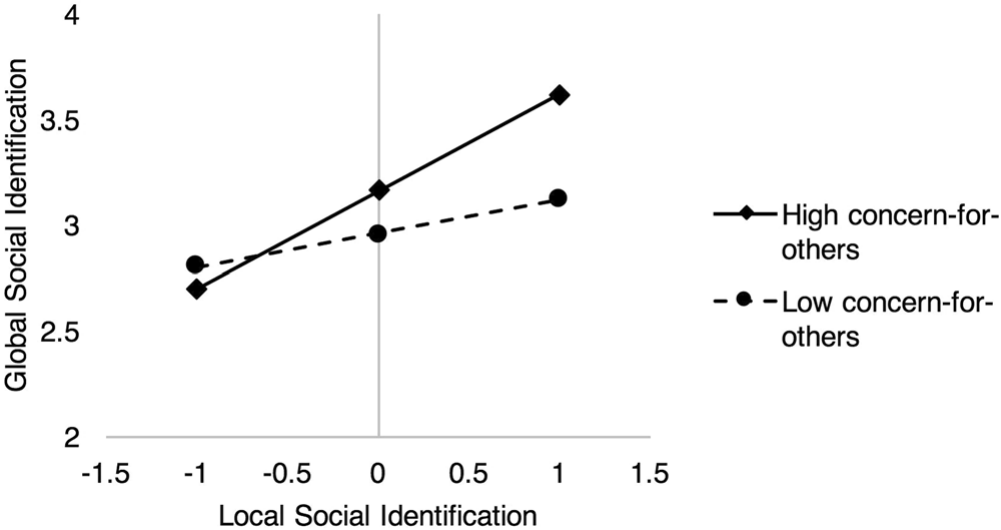
Best-fitting regression lines showing the effect of local social identification on global social identification when concern-for-others is high versus low. Estimated slopes at +/− 1 s.d. around the mean of concern-for-others are shown. Simple slopes analysis for the interaction between local social identification and concern for others on global social identification showed that for individuals who were high in concern-for-others, the simple slope of local social identification was positive (*b* = 0.46, *SE* = 0.10, 95% CI = [0.27, 0.65], *t*(167) = 4.84, *p* < 0.001), whereas the simple slope was non-significant (*b* = 0.16, *SE* = 0.11, 95% CI = [−0.06, 0.37], *t*(167) = 1.43, *p* = 0.156) for those low in concern-for-others.

We then included the interaction term between concern-for-others and local social identification (adjusted R^2^ = 0.18, *F*(5, 167) = 7.49, *p* < 0.001; delta R^2^ = 0.02, *F*(5, 167) = 4.01, *p* = 0.047). The pattern of simple slopes (see Fig. 1) for the interaction term (*b* = 0.15, *SE* = 0.08, 95% CI = [0.002, 0.304], *t*(167) = 2.00, *p* = 0.047) showed that, for individuals who were high in concern-for-others, the relationship between local and global social identification was positive (*b* = 0.46, *SE* = 0.10, 95% CI = [0.27, 0.65], *t*(167) = 4.84, *p* < 0.001). However, for individuals who were low in concern-for-others, no such relationship was found (*b* = 0.16, *SE* = 0.11, 95% CI = [−0.06, 0.37], *t*(167) = 1.43, *p* = 0.156). The direct effect of local social identification on global social identification was also positive in the presence of the interaction (*b* = 0.31, *SE* = 0.07, 95% CI = [0.17, 0.45], *t*(167) = 4.48, *p* < 0.001). Thus, local social identification predicted global social identification to a greater extent for those who were high in concern-for-others, whereas the relationship was non-significant for those who were low in concern-for-others.

We tested for internal replication of these findings by running the same model within each country. The analyses produced simple slopes consistent with our findings for both South Korean and US samples where the relationship between local social identification and global social identification was positive for high concern-for-others individuals (US: *b* = 0.30, *SE* = 0.12, 95% CI = [0.07, 0.53], *t*(91) = 2.57, *p* = 0.012; South Korea: *b* = 0.83, *SE* = 0.17, 95% CI = [0.49, 1.18], *t*(72) = 4.81, *p* < 0.001). Furthermore, also consistent with the findings, the relationship between local social identification and global social identification was weaker for low concern-for-others individuals for both samples, (US: *b* = 0.14, *SE* = 0.12, 95% CI = [−0.11, 0.38], *t*(91) = 1.11, *p* = 0.272; South Korea: *b* = 0.19, *SE* = 0.26, 95% CI = [−0.32, 0.70], *t*(72) = 0.75, *p* = 0.454).

### Global Contribution

We further explored with the full two-country sample whether the extension of local social identification to global social identification translates into cooperative behavior at the global level. Consistent with findings from Buchan *et al*.^12^, a multiple regression analysis (adjusted R^2^ = 0.06, *F*(4, 168) = 2.76, *p* = 0.029) showed that global social identification predicted global contribution (*b* = 0.59, *SE* = 0.24, 95% CI = [0.11, 1.07], *t*(168) = 2.43, *p* = 0.016), even when country, income, and local social identification were included as covariates.

**Figure 2:**
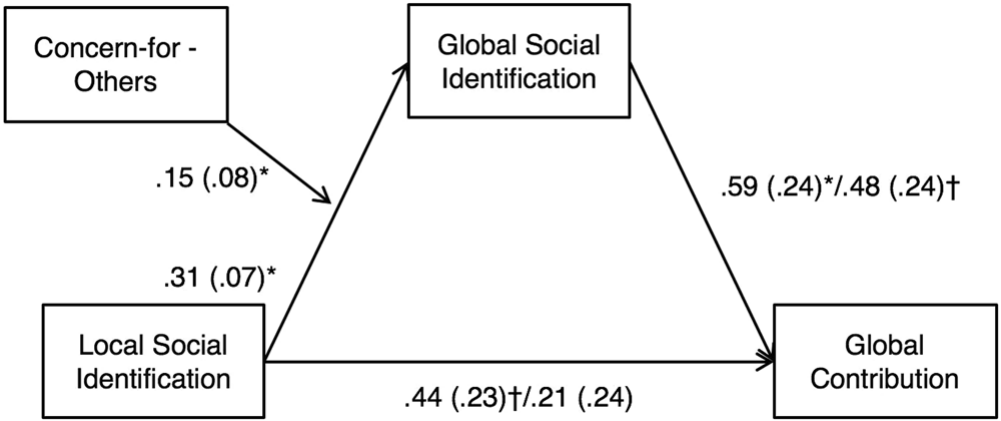
Moderated mediation model of the effect of local social identification on global contribution as mediated by global social identification and moderated by concern-for-others. Unstandardized regression coefficients are reported, with standard errors in parentheses. Coefficients to the right of the slash are simultaneous regression coefficients. ^*^ *p* < 0.050, ^†^ *p* < 0.100, two-tailed. The interaction between local social identification and concern for others on global social identification was significant (*b* = 0.15, *SE* = 0.08, 95% CI = [0.002, 0.304], *t*(167) = 2.00, *p* = 0.047), which, in turn positively related to global contribution (*b* = 0.59, *SE* = 0.24, 95% CI = [0.11, 1.07], *t*(168) = 2.43, *p* = 0.016). The direct effect of local social identification (*b* = 0.44, *SE* = 0.23, 95% CI = [−0.235, 0.716], *t*(169) = 1.93, *p* = 0.056) became weaker and non-significant in the simultaneous model (*b* = 0.21, *SE* = 0.24, 95% CI = [−0.260, 0.686], *t*(166) = 0.89, *p* > 0.250), whereas the direct effect of global social identification remained marginally significant (*b* = 0.48, *SE* = 0.25, 95% CI = [−0.004, 0.967], *t*(166) = 1.96, *p* = 0.052).

The findings so far suggest that local social identification predicts global social identification (more so for those who are high in concern-for-others), and that global social identification, in turn, predicts global contribution. Figure 2 depicts the model and shows the summary of coefficients from the regression analyses.

### Test of Moderated Mediation

These relationships can also be validated through a test of moderated mediation. We further explored if the indirect effect of local social identification on global contribution through global social identification was also contingent on concern-for-others. We followed Hayes and Scharkow^25^ to produce a bias-corrected 95% confidence interval for the indirect effect from 5,000 bootstrap samples using the PROCESS macro^26^. We included country and income as covariates, and set local social identification as the independent variable, global social identification as the mediator, concern-for-others as the moderator for the first stage, and global contribution as the dependent variable.

**Figure 3:**
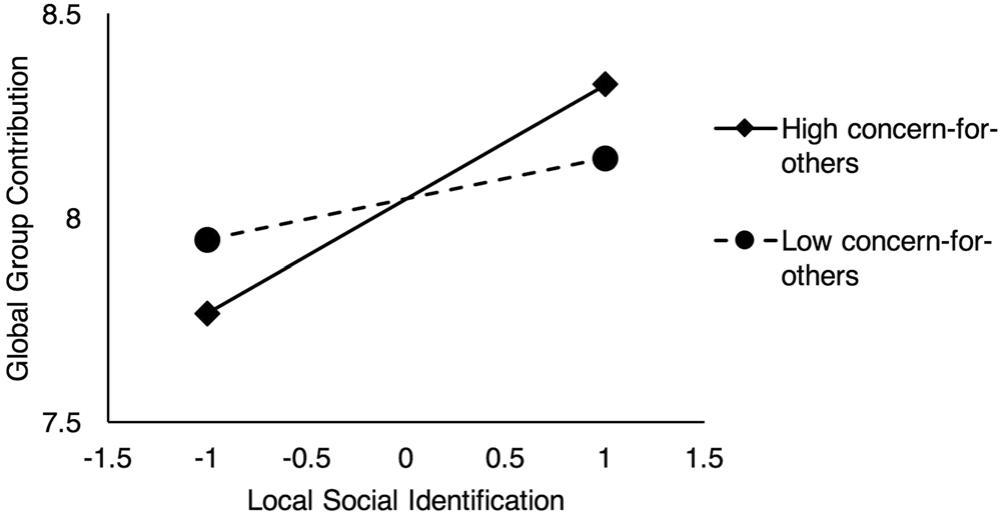
Best-fitting regression lines showing the conditional indirect effect of local social identification on global contribution through global social identification when concern-for-others is high and low. Estimated slopes at +/−1 s.d. around the mean of concern-for-others are shown. For individuals who were high in concern-for-others, the indirect effect of local social identification on global group contribution through global social identification was positive (*b* = 0.28, *SE* (Boot) = 0.13, 95% bias-corrected CI = [0.066, 0.577]), whereas the indirect effect was non-significant for those low in concern-for-others (b = 0.10, SE (Boot) = 0.08, 95% bias-corrected CI = [−0.009, 0.315]).

The results indicated a moderated mediation effect (index of moderated mediation = 0.09, *SE*(Boot) = 0.06, 95% CI [0.003, 0.250]). When concern-for-others was high (1 SD above the mean), the conditional indirect effect of local social identification on global contribution through global social identification was positive (*b* = 0.28, *SE* (Boot) = 0.13, 95% bias-corrected CI = [0.066, 0.577]). When concern-for-others was low (1 SD below the mean), there was no conditional indirect effect (*b* = 0.10, *SE* (Boot) = 0.08, 95% bias-corrected CI = [−0.009, 0.315]). Figure 3 illustrates the simple slopes of the conditional indirect effect at high and low levels of concern-for-others. As expected, the direct effect of local social identification on global contribution was nonsignificant (*b* = 0.24, *SE* = 0.24, 95% CI = [−0.235, 0.716], *p* = 0.320).

## Discussion

The results of our research suggest that social identification at a local level can be extended to the global community resulting in cooperative behavior with a distal global collective. Moreover, we found that concern-for-others aids in this identification extension. Among participants who were low in concern-for-others, identification with the local group was unrelated to identification with the global community and to contribution to the global group. That is, when lacking concern-for-others, identification with the local group did not extend beyond the boundaries of their in-group. Among participants high in concern-for-others, higher local social identification was linked to higher identification with the global community, and in turn, positively predicted global group contribution. In other words, concern-for-others appears to enhance identity extension, pushing individuals past parochial boundaries, and leading to global cooperation. These findings shed new light on the role of concern-for-others, in that in addition to the well-documented direct effect on cooperative behavior (which was also found in the current study; *r* = 0.13, *p* = 0.078), concern-for-others prompts locally-identifying individuals to cooperate globally through global identification.

Theoretically, these results are consistent with Gaertner *et al*.^15^ who demonstrated that generalization of identity is maximized when the salience of the initial group identification is maintained within the context of a salient common in-group supraordinate identification. This suggests that strong identification with the local community need not be in opposition to more expansive global identification; one can identify both locally and globally. The current exploration, however, submits that such generalization is more likely to occur among those individuals who are high, rather than low, in concern-for-others. In other words, an additive (as opposed to conjunctive) identification strategy seems to be compatible with the trait concern-for-others, as individuals high in concern-for-others were more likely to extend their local identification to global identification.

The impact of concern-for-others on identity extension may be explained by a deeper understanding of the motivations for group identification^27^. It may be that for individuals high in concern-for-others, the motive for identity extension is governed by inclusion/assimilation needs, that is, the need to get along. Because individuals high in concern-for-others desire a sense of belonging and view group membership through the lens of connectedness^22–24^, they may be more likely to use an additive strategy and view themselves dually as local and global group members. On the other hand, individuals low in concern-for-others may be driven by their need for differentiation/distinctiveness from others. The introduction of a “global community” may challenge the distinctiveness of their parochial group and, indeed, may threaten cultural values that are central to members’ functioning^16^. As such, a more expansive identity may be resisted in favor of a conjunctive strategy where identification with the local group does not extend to identification with the greater global collective.

It is interesting to note that our findings held in two countries that are known to vary in the manner in which people tend to relate to others. Individuals from South Korea tend to show stronger focus on tight in-group relationships (such as with family members) than do individuals from the United States^28^, suggesting that extension of identification may be more difficult to achieve in this society. However, our results suggest an identification extension effect in samples in both countries, demonstrating initial evidence of universality of the underlying psychological mechanisms of the phenomenon.

Our findings suggest that it would behoove researchers interested in cooperation with larger or distal collectives to focus on understanding antecedents, such as concern-for-others, that might aid in extension of identification. Future research may explore other personality traits and characteristics that also have a qualifying effect for identity extension. Further, concern-for-others is thought to represent both an individual disposition and a motivational state^29^. While in this study we employed trait concern-for-others as a relatively stable individual difference, others have used experimental priming to induce other-oriented mindset^30,31^. This line of research suggests malleability of concern-for-others, which, according to our results, suggests malleability of degree of identification extension.

The study is not without limitations. Firstly, variable measurement was cross-sectional. Therefore, drawing causal inference from the current study should be done with caution. Supplemental analyses demonstrated that including other potential third variables such as concerns for reputation (captured by the number of strangers in the local community), expected contribution by other members, social desirability, and local contribution in our model did not alter the pattern of significant results. Analyses revealed that the scale concern-for-others did have a low to moderate correlation with similar variables such as empathic concern (*r* = 0.166, *p* = 0.029)^32^ and social value orientation (*r* = 0.25, *p* = 0.007)^33^. Yet, inclusion of empathic concern and social value orientation as covariates did not alter the pattern of significant results, nor did models substituting each of these variables for concern-for-others produce the same pattern of results. The low to moderate correlation coefficients suggest that these variables reflecting prosocial tendencies are similar to concern-for-others. Yet, it is also clear that concern-for-others explains unique variance in global cooperation and that the other variables do not help translate identity extension into actions of global cooperation.

Second, although we did collect data from two different countries, and despite demonstrating internal replication with the two samples, external validity questions still apply. The church-member sample used in this study may have demonstrated higher levels of prosociality and norms/cultures of altruism than the general population. However, such characteristics would have restricted the variance in the key variables which would have created a more conservative ground for our findings to be detected. Furthermore, even if our sample were more altruistic and cooperative than the general public, these tendencies would have strengthened the direct effect of concern-for-others on cooperation, not the moderating role of concern-for-others on cooperation with global others (the central finding of this research).

Notwithstanding the limitations, our findings are especially encouraging given the national, regional, and demographic divisions that have recently surfaced so dramatically. Since we have demonstrated the possibility of extending locally-based identification to a larger collective, the suggested first step for public policy makers addressing global challenges may be to build identification within local communities in which decisions for global cooperation take place. Individuals who have strong identification to the local collective, and who are high in traits such as concern-for-others, are likely to break the boundaries of parochialism and extend identification and helpful behavior – at an expense to themselves – to anonymous strangers in other parts of the world.

## Methods

This study was approved by the institutional review board (IRB) of the University of South Carolina. All methods were performed in accordance with the IRB guidelines and regulations. Informed consent was obtained from all study participants prior to data collection.

### Sample

Our research questions called for a context with pre-existing membership to a local community, with a varying degree of identification to the local group. We chose Christian churches based in the US and South Korea to recruit participants, as they provided a local community context within which individuals have the discretion to join and maintain regular membership. This design allowed us to hold the nature of the local community (a religious group) constant while collecting data from two distinct cultures. Two churches in each country were recruited with the promise of receiving a $100 donation, and individual participants were recruited within churches with the promise of $4 for attending the session and an additional average of $20 per person resulting from payoffs of the game.

We used G*Power 3.1 software to determine the required sample size to detect at least a medium effect size (similar to those detected in past identity studies)^7,13^ with a t-test or Cohen’s d at a 0.05 α level^34^. Results showed that 111 participants would be required to detect a *t* of 1.66, and 54 would be required to detect a *d* of 0.50. We therefore recruited in excess of these numbers and were able to collect data from 194 individuals. Analyses excluded data from 21 participants (12 Korean, nine American) who failed a comprehension check, leaving a total of 173 participants (77 Koreans, 96 Americans).

We included country (0 = US, 1 = South Korea) and annual household income as covariates to account for differences associated with national origin and income level. There were no differences on key variables nor was there an interaction between country and the social identification measures. There was no meaningful difference between the average giving in the two countries, on either global contribution (*M*_South Korean_ = 6.72, *SD*_South Korean_ = 2.91, *M*_US_ = 7.21, *SD*_US_ = 3.12; *t*(171) = −1.06, *p* = 0.289) or local contribution (*M*_South Korean_ = 6.99, *SD*_South Korean_ = 2.82, *M*_US_ = 7.23, *SD*_US_ = 3.00; *t*(171) = −0.55, *p* = 0.583). We also found that neither the significance nor the pattern of results was altered by inclusion of gender, local contribution, expected contributions from others, social value orientation, or empathic concern in the model. To avoid over-fitting the model, we excluded these variables from our analyses. We standardized all predictor variables to ease the interpretation of simple slopes and to address potential multicollinearity issues.

### Procedures

We addressed our research questions in the context of a public goods dilemma^35^. Seventeen sessions (eight in South Korea, nine in the United States, with a minimum of four participants each) were conducted in the local language at participants’ respective churches. To ensure cross-country comparability of the data sets, instructions and procedures were standardized across countries and scripts were translated and back-translated for consistency. To ensure confidentiality, physical privacy barriers were installed between participants.

Participants who arrived at the session were given their show-up fee and were informed at the beginning of each session that they would be asked to make two decisions. They were told that for each decision, the final outcome/payment would depend on their own decisions, as well as on those of three others to whom they were matched by computer algorithm. Participants were further informed that in the first decision (involving a local pot), the three other participants would be from their local church (but perhaps not in the same room), and that in the second decision (involving a global pot), the three other participants would be from around the world. The local decision was introduced to make the local community context more salient to the participants and to set the stage for the global decision. The identities of the other participants were anonymous in each decision.

Participants were given 10 tokens for each decision, and each token was worth the purchasing-power equivalent of $0.50. The value of tokens participants kept for themselves stayed constant, but each token shared with the group pot (whether local or global) was multiplied by two and shared among the whole group. A participant who gave nothing to the group account would retain all ten tokens. However, if all participants contributed all of their tokens to the group pot, each participant would receive 20 tokens. The maximum pay-out would come to a participant who kept all ten tokens but whose three group members allocated all tokens to the group, resulting in 10 + 15 = 25 tokens. Participants were not given feedback between local and global decisions. A post-game questionnaire including items measuring local and global social identification as well as concern-for-others was administered after the participants finalized their decisions.

Because of the logistical difficulty of conducting the experiment simultaneously in multiple sessions within different countries, we relied on a dynamic matching algortithm where past participants’ decisions were used to determine the payoffs of current participants. The decisions of current participants were entered into the dataset, and earnings computed by the algorithm, while they completed their post-study survey. Participants’ outcomes were determined by their decision and those made by arbitrarily selected groups of participants either from the participant’s church or from another country around the world. Participants received payoffs in an envelope and were debriefed at the end of the experiment as they walked out of the study venue individually, thus no feedback was provided regarding decisions during the session, nor was it made public to other participants.

### Measures

*Global group contribution* was measured with the number of tokens a participant placed in the global pot. Items for both *local social identification* and *global social identification* were adapted from Buchan *et al*.^12^. Item stems were “How strongly do you feel attachment to…,” “How strongly do you define yourself as a member of…,” and “How close do you feel to other members of…” Each stem was followed by either “…your [church name] community?” to assess local identification, or “…the world as a whole?,” to assess global identification. Response options ranged from 1 = “not at all” to 5 = “very strongly.” Cronbach’s alphas were 0.86 and 0.88 for local and global social identification measures, respectively.

We assessed *concern-for-others* with the concern-for-others subscale in Ravlin and Meglino’s^36^ Comparative Emphasis Scale (CES). CES presents 24 pairs of statements and asks the respondents to choose a statement they think is more important. CES is distinct from similar other-orientation measures (for example, empathic concern) in that it is an ipsative measure forcing respondents to choose between equally attractive options. That is, respondents are required to choose between pairs of statements representing one of four values (concern-for-others, fairness, achievement, and honesty-integrity), each matched for social desirability, reducing measurement errors associated with normative measure. Another important strength of CES is that it addresses the hierarchical nature of values^37^, allowing us to assess the importance of concern-for-others relative to other values. The validity of concern-for-others subscale of CES has been demonstrated in previous research. We scored the 12 statements assessing concern-for-others to create a rating ranging from zero to twelve, reflecting the number of times a participant indicated preference for a statement representing concern-for-others over a statement representing another value. Sample concern-for-others statements include “Trying to help someone through a difficult time,” or “Trying to help reduce a friend’s burden”. These items were paired with items representing another desirable value such as “Being impartial in dealing with others” (fairness) and “Speaking your mind even when your views may not be popular” (honesty-integrity).

As mentioned in the results section, concern-for-others was positively correlated with social value orientation and empathic concern. Social value orientation and empathic concern were positively correlated with global contribution (social value orientation, *r* = 0.15, *p* = 0.079; empathic concern, *r* = 0.18, *p* = 0.018). These findings are consistent with the moderate correlations reported in previous studies with other measures of other-oriented variables^38,39^, Also, concern-for-others was not correlated with expected contributions (the number of tokens the participants expected the other three players to put in the global pot; *r* = 0.007, *p* = 0.922), which could be an indicator of conditional cooperation.

### Code availability

The SAS codes are available to the public on Open Science Framework.

### Data availability

All data, statistical analyses, and associated Figures (1, 2 and 3) that support the findings of this study are available to the public on Open Science Framework.

## Electronic supplementary material

Supplementary information
