# Supplementary material for: Local Reasons to Give Globally: Identity Extension and Global Cooperation
Source: Sci Rep. 2017 Nov 14;7:15527. doi: 10.1038/s41598-017-15683-0 (PMC5686204; doi:10.1038/s41598-017-15683-0)
Supplement: Supplementary file 1 — Supplementary information [file 41598_2017_15683_MOESM1_ESM.pdf]

Local Reasons to Give Globally: Identity Extension and Global Cooperation

Nancy R. Buchan[1], Sophia Soyoung Jeong[2]*, AK Ward[3]

[1] Department of International Business, Darla Moore School of Business, University of South Carolina

[2] Management Department, Faculty of Business Administration, Chinese University of Hong Kong

[3] Department of Management, Pamplin College of Business, Virginia Tech University

*Correspondents should be addressed to Sophia Soyoung Jeong, Management Department, Chinese Faculty of Business Administration, Chinese University of Hong Kong, 12 Chak Cheung Street, Sha Tin, N. T. Hong Kong, sophia.jeong@cuhk.edu.hk

Supplementary Information

Experimenter Script for Public Goods Games

Note: *Instructions to experimenters are in italics. Text in non-italics is read to the Ss. Hereafter RA refers to the Research Assistant*

As subjects enter the room, ask them to please sit wherever there is a box and read/sign the informed consent form. RA will collect informed consent forms as they are completed.

Once all subjects have handed in their informed consent forms, RA will give them their show-up fee envelopes. She will also hand out ID # sticker shoots, allowing each participant to randomly choose his/her own.

Please turn off your cell phones. Please do not text or talk to anyone in this room until the experiment is over.

First, please take your show-up fee out of the envelope and put it away. This money is yours to keep for coming to the experiment. Next, please look at your sheet of stickers. These stickers will have your ID number on them. You will notice you have 12 marked stickers on the sheet. These will be used later for your decisions in the experiment; you will put the ID stickers in the upper right corner of different pieces of paper and on envelopes. You will need to have at least 1 sticker left at the end so we may match you to your payment at the end of the experiment, so please do not waste your stickers.

Now, please place one ID sticker on the empty envelope. We will collect these envelopes now, and at the end of the experiment each envelope will be returned – containing the experiment earnings – to the person with the matching ID number. We will not know who you are or what decisions you made – we will only know your ID number.

RA will collect the envelopes.

Please turn your sheet over and do not show it to the others. Do not tell anyone, except the experimenter, your number. **Please keep quiet and do not speak to the others in this room.**

The instructions can now begin.

Welcome to this research project. An international team of researchers is looking at the way in which people in this community and around the world make decisions. If you pay close attention to the instructions then you could make a significant amount of money.

My name is ________, and I am here to explain the rules of this project to you. The research team that is here today includes the Research Assistant ______ and Professor ______ from University ______. For most of this experiment, Professor _______ will be outside the room

Everyone should be present in the room at this point and acknowledge the introduction. Professor ______ should then leave.

In this project, you are going to be asked to make decisions with other people. Some will be from this church, but maybe not in this room; some will be from countries around the world. Many people have already made their decisions, and other groups have done the same research this summer. Your choices, and the choices by others, will be matched using a computerized algorithm when you are finished. You will be paid in cash at the end of this research for the decisions that you and the people you have been matched with made. **The money is yours to keep.**

The same instructions have been given to other people in other countries. That is why we are reading this script. Everyone has heard the same thing you are hearing, except in their own language.

All of the decisions are similar, so please pay attention to these instructions. At the outset of each decision you will be given 10 colored tokens. Everyone will get the same materials that you get. It will be important to keep in mind that colored tokens are worth 50 cents each to you. For other people, whether from ______ Church or another part of the world, their colored tokens also are worth money to them. We have taken care that their tokens, once converted to their foreign currency, are worth the same value as your tokens in terms of what could be purchased with them. That is, people in other countries will receive an amount in their currency such that they can buy in their country the same amount of goods that 50 cents will buy in the United States.

Again, keep in mind that you are being matched with other people (some of whom are from _______ Church and some of whom are from around the world). What those people have decided to do and what you will decide to do affects how much you can make. When your decisions are submitted, Professor __________ will be using our computer connection to receive information about others' choices in order to calculate each person's payments. This may take a little while, so please be prepared to wait for a few minutes at the end of the session so that we can give you your final payment before you leave today.

After a pause, begin the instructions...

Your task is to decide how you want to allocate your tokens between different envelopes. You will have two options for each decision, and you will have one decision to make with each of two groups. I will first explain the basic rules of the game, then give you information about who will be in your first decision group.

Before each of the two decisions, you will be given 10 tokens. You can put your tokens into your "Personal" envelope or into your group envelope. The number of tokens you put into either envelope is entirely up to you.

What's the difference between the envelopes? Whatever you put into the "Personal" envelope [illegible]

Now, what about the group envelope? Any colored tokens that you and three other people put into your group envelopes will be doubled by the researchers. You and the other three people will get an equal share of that amount.

At this point, RA should pass out the example sheet.

EXAMPLES.

Before we begin the decisions, I want to make certain you understand how you get paid. Please make certain you know exactly how you can receive money. You will be paid based on the decisions that you and the others you are mixed with make.

Please follow along with the examples that have been handed out to you. We will now go through them each together. For example #1, suppose that you put 10 yellow tokens into your “Personal” envelope and the other three people put a total of 12 yellow tokens in their group envelopes. In that case, the 12 tokens in the group pot will be doubled (to 24) and shared equally among you and the other three people (6 each).

You would then receive a total of 16 tokens: 10 from your “Personal” envelope that you kept, and 6 from your share of the group pot; given that there were 12 yellow tokens in the group envelopes, that amount would be doubled to 24 by the researchers and you would get an equal share, which is 6 yellow tokens. You would end up with 16 tokens worth $8.00. Is anyone uncertain about how this happens?

To take another simple example (#2), suppose you put 8 of your yellow tokens in the group envelope and no one else put any yellow tokens in the group envelope. What would you receive? If you like you can write in the blanks on the example.

Wait while participants make calculations; look around to see if they are attempting to come up with the answer; encourage someone to give an answer.

You would receive a total of 6 tokens. First, you would have 2 yellow tokens in your “Personal” envelope. Given that there were 8 yellow tokens in the group envelopes (all put there by you), that amount would be doubled to 16 and you would get an equal share, which is 4 yellow tokens. The other people in your group also get 4 yellow tokens. You would end up with 6 tokens worth $3.00.

Finally, let me give one more example (#3). Suppose you put all 10 of your yellow tokens in the group envelope. Suppose that the other 3 people did the same thing. That means there is a total of 40 yellow tokens in the group envelopes. How much would you receive?

Wait for an answer from participants...

You (and the other three people) would receive a total of 20 tokens, which is $10.00 for this decision. First, you would have 0 yellow tokens in your “Personal” envelope. Second, in the group envelopes there would be 40 tokens. This would be doubled to 80 and your share would be 20 tokens.

These three examples show that what you get can be very different, depending both on what you and everyone else does. Please take the time to look through the examples. Do this while we are passing out a new sheet of paper.

At this point, RA will pass out the comprehension sheet.

A sheet is being handed out to you with three questions on it. Please do not answer the questions until I read them aloud.

When the sheets are handed out, begin the instructions.

Before you do anything, please remove one of your stickers and put it in the upper right hand corner of the sheet that was just handed out.

Pause until everyone has done so.

I am going to read the questions one at a time. Please check the answer you think is most appropriate. When everyone is done I will read the answers.

1. What happens when a token is put into the group envelope? (Nothing; The token is cut in half; The token is doubled; The token is tripled)

2. How many people, including you, are in the group? (Two people; Three people; Four people; Five people)

3. Everyone gets an equal share of the group envelope. (True or False?)

Pause until everyone is finished.

Now that you are done, let's go over the answers. Do not mark your papers. They will be collected later.

1. What happens when a token is put into the group envelope? The token is doubled and you get an equal share along with the other people in the group.

2. How many people, including you, are in the group? There are a total of four people in the group. This includes you. This means each of you will get a one-quarter share of the tokens that are put into the envelopes and doubled.

3. Everyone gets an equal share of the group envelope. This is true – everyone gets an equal share.

Do you have any questions? RA will now collect your comprehension sheets and pass out some materials. **Please do not do anything with the envelopes until I instruct you to do so!!!**

At this point, RA will collect the comprehension sheets (making sure there is an ID sticker on each) and pass out all decision 1 materials (local/personal envelopes, tokens, purple

`

DECISION ONE.

Please do not divide your tokens between the envelopes until I instruct you to do so.

Now, let me tell you about who is in your group for the first decision. You should have an envelope marked "Personal" and an envelope marked "______ Church." You should have 10 yellow tokens (each of which are worth 50 cents to you and everyone else). Your tokens are in your "Personal" envelope. Please take them out and count them to make certain that you have 10. *(Give them a minute to count).* Please raise your hand if you have too many tokens or if you need more.

For the ______ Church decision in this experiment you will be matched with three other people from this church. They may or may not be in this room. I do not know which people you will make decisions with because you will be mixed anonymously with other people in order to make a group of four. All of the people you are mixed with are from _______ Church.

The first thing I would like you to do is to take stickers from your ID card and put them on the upper right corner of both your envelopes and your purple paper. Please make certain you do this. This is the only way we can make certain you will be paid.

At this point the experimenter can demonstrate how this is done on a blank envelope.

Also, it is important that you do not write on, fold, or damage the envelopes in any way. Only your ID sticker should be on the envelope.

You can put any combination of tokens into the 2 envelopes. Remember that the yellow tokens you put into your "Personal" envelope are yours and will not be divided among any others. Whatever you and the three other participants from ________ Church put into the group envelopes will be doubled. Each of you will get an equal share of that amount.

When you have made your decision, place the envelopes on top of your box. DO NOT seal the envelopes. RA will come around and collect your envelopes. She will check to make certain you have put your ID number in the upper right corner of your envelopes. When you have finished, put your envelopes on top of your box so we will know you are finished. If you have any questions please raise your hand.

RA will collect all personal and _______ envelopes in separate "Decision 1" boxes. She will double-check each envelope to make certain that it has an ID number attached to it. If not, she will ask the subject to do it before the envelopes are placed in the boxes.

RA will take the envelopes to Professor _______, who is outside the room. Professor ______should open the personal envelopes and enter and record the number of yellow tokens for each subject. She should then open the group envelope, enter and record the number of yellow tokens, check the group assignment and calculate the [illegible]

The data for each participant's choices and payment will also be entered onto the session spreadsheet.

Now that everyone's decision has been made, the envelopes will be matched with other people and how much money you receive will be calculated. It will take a while to do this. At the end of the session you will be given an envelope with your payment.

While we prepare for the next round, please take a moment to write on the purple paper provided about whatever is on your mind right now.

Time 2 minutes for them to write. RA will return before the 2 minutes are up and will collect all sheets, making sure ID stickers are on all sheets.

*RA will hand out the decision 2 bundle of materials (personal/global envelopes, tokens**;** Ss each get a bundle.*

Please do not divide your tokens between the envelopes until I instruct you to do so.

DECISION TWO.

The second decision follows the same rules but involves a different group of people. In this decision, you will have 10 blue tokens and 2 envelopes. Once again you will be paid 50 cents for each blue (colored) token. Please take them out and count them to make certain that you have 10. *(Give them a minute to count).* Please raise your hand if you have too many tokens or if you need more.

As with the first decision, the blue tokens you put in your "Personal" envelope will be yours and not divided with anyone else. Second, the blue tokens you and the others put into the "Global" envelope will be doubled and you will get an equal share from the doubled amount.

For the GLOBAL decision in this experiment you will be matched with three other people from other parts of the world, including Asia, Africa, Europe, and North or South America. The team has collected decisions made by other people who have faced the same choices as you, and they have sent their results back to a central administrator who will tell us what different groups have decided to do. Your decision will be randomly mixed with what other people have done and this will determine your final payment. Your decisions will also affect the payments of the others in your group.

Please remove ID stickers and put one on each of the two envelopes. Please do this now and make certain it is in the upper right corner.

You can put any combination of tokens into the 2 envelopes. Remember that the blue tokens you put into your "Personal" envelope are yours and will not be divided among any others. Whatever you and the three other people from around the world put into the "GLOBAL" envelopes will be doubled. Each of you will get an equal share of that amount.

After you make your decision, please DO NOT seal the envelopes, and put the materials on

certain you have put your ID number in the upper right corner of both of your envelopes. When you are finished, please put all of your materials on top of your box.

RA will collect all personal and global envelopes in separate "Decision 2" boxes. She will double-check each envelope to make certain that it has an ID number attached to it. If not, she will ask the subject to do it before the envelopes are placed in the boxes.

RA will take the envelopes to Professor ______, who is outside the room. Professor _______ should open the personal envelopes and enter and record the number of blue tokens for each subject. She should then open the group envelope, enter and record the number of blue tokens, check the group assignment and calculate the share obtained by the S. The personal tokens and the Ss group share will be filled out on the decision record slip of paper for each participant.

Professor ______ then combines information from both decisions for each participant and calculates their total payment, which is recorded on the decision slip for that participant (and on the session log sheet). The decision slip is put into an envelope, along with the total payment in money (rounded to the nearest whole number) and labeled with the participant ID number.

Now that everyone's decision has been made, the envelopes will be matched with other people and how much you money you receive will be calculated. It will take a while to do this.

You have now finished making your decisions with others. Before you are given the payments from your decisions, I am going to pass out a questionnaire. This questionnaire will help us get more information about the people who participated in the decisions. Your questionnaire will have only your ID number. We will not know who you are and how you responded. Please be as honest as you can with your answers.

The reader passes out the questionnaire, with the expectancy measures first.

Please take a sticker off of your ID card and put it in the upper right corner of the first page of the questionnaire you have been handed. When you are finished, please place your questionnaire and pen on top of your desk. Once everyone is finished, we will wait while the payment calculations are being completed.

When all participants are finished they can be brought out to Professor __________ one at a time, with their questionnaire. Professor ______ will then give the participant the payment envelope which matches the participant's ID#.

Participants should then count and verify that they have received the amount listed on the decision slip which is in the envelope, and Professor ______ will fill in the participant's ID number on the log sheet, the amount received, and then put his/her initials next to it. Professor _______ will then cover that particular line of the log sheet and the next participant will come in.
